# Graduate Courses at the Infants Hospital

**Published:** 1908-12-12

**Authors:** 


### GRADUATE COURSES AT THE INFANTS HOSPITAL.

The Medical Committee of the Infants Hospital, Vincent Square, Westminster, have been authorised to make arrangements whereby medical practitioners may obtain systematic instruction in the modern scientific methods relating to infantile dietetics and the treatment of the nutritional disorders of infancy. The proposed classes will each consist of six demonstrations to be completed in the course of a fortnight—three in each week, on Tuesdays, Thursdays, and Fridays, from about 4.30 to 6 p.m. The course will be essentially practical and clinical. Three of the demonstrations will be conducted in the research laboratory, when each member of the class will be able to carry out the experiments individually. These experiments, connected with the chemistry of milk in its relation to infantile digestion, will be arranged so as to have a direct bearing on the ordering and preparation of foods for infants and the treatment of their nutritional disorders. The remaining demonstrations will be clinical, and will deal with the treatment of the diseases and disorders of nutrition, dietetic and otherwise. The classes will be limited in number. The fee for the course will be one guinea, which will include the use of all apparatus, etc. It is proposed to begin the first class in the early part of 1909, when further details will be announced. The classes will be confined strictly to qualified medical practitioners; those desirous of joining are requested to communicate with the Hon. Secretary of the Medical Committee without delay. The first course will be given by Dr. Ralph Vincent.

